# The Influence of Premature Birth on the Development of Pulmonary Diseases: Focus on the Microbiome

**DOI:** 10.3390/metabo14070382

**Published:** 2024-07-11

**Authors:** Magdalena Wolska, Tomasz Piotr Wypych, Pilar Rodríguez-Viso

**Affiliations:** Laboratory of Host-Microbiota Interactions, Nencki Institute of Experimental Biology, Polish Academy of Sciences, Ludwika Pasteura 3, 02-093 Warsaw, Poland; m.wolska@nencki.edu.pl (M.W.); p.rodriguez-viso@nencki.edu.pl (P.R.-V.)

**Keywords:** preterm, gut microbiome, immune system, antibiotic, asthma, chronic obstructive pulmonary disease

## Abstract

Globally, around 11% of neonates are born prematurely, comprising a highly vulnerable population with a myriad of health problems. Premature births are often accompanied by an underdeveloped immune system biased towards a Th2 phenotype and microbiota dysbiosis. Typically, a healthy gut microbiota interacts with the host, driving the proper maturation of the host immunity. However, factors like cesarean section, formula milk feeding, hospitalization in neonatal intensive care units (NICU), and routine antibiotic treatments compromise microbial colonization and increase the risk of developing related diseases. This, along with alterations in the innate immune system, could predispose the neonates to the development of respiratory diseases later in life. Currently, therapeutic strategies are mainly focused on restoring gut microbiota composition using probiotics and prebiotics. Understanding the interactions between the gut microbiota and the immature immune system in premature neonates could help to develop novel therapeutic strategies for treating or preventing gut–lung axis disorders.

## 1. Introduction

Preterm birth affects approximately 11% of births worldwide [1] and can either occur spontaneously or be induced by infections or pregnancy complications that require cesarean delivery. Premature neonates are classified as extremely preterm (<28 weeks of gestational age, GA), very preterm (28–32 weeks of GA), and moderate to late preterm (32–37 weeks of GA) [2]. Premature births result in impaired intestinal physiology and immune deficiencies, including lymphopenia, altered balance of Th1/Th2 responses, increased Th17 and Treg populations, and reduced numbers of neutrophils and NK cells accompanied by dysfunctional dendritic cells. Such changes create an immune system incapable of mounting adequate immune responses. Moreover, preterm infants are hospitalized for the first days in the neonatal intensive care units (NICU), where they receive antibiotics, are often fed with formula-based milk, and have limited exposure to the mother’s microbes [3]. All these factors make the gut more susceptible to disrupted colonization, represented by potentially pathogenic facultative anaerobic Gammaproteobacteria and strict anaerobes [4], putting the infants at a major risk of developing enteric infections. The gut microbiota also modulates immune responses in the airways via the “gut–lung axis” [5], and gut dysbiosis in early life may lead to an increased incidence of developing pulmonary disorders later in life [6].

Currently, several microbiota-targeting treatments have shown the potential to improve dysbiosis in preterm children. For instance, probiotic [7] and prebiotic supplementation [8] are easily available and have been shown to improve health outcomes in premature neonates. For instance, the administration of *Lactobacillus* and *Bifidobacterium* species, and human milk oligosaccharides modulate preterm gut microbiota, increasing the proportion of *Bifidobacterium* species among that of facultative anaerobe and pathogenic species [9,10]. Moreover, the supplementation of these bacterial species increases the content of short-chain fatty acids (SCFA), indicating that *Bifidobacterium* can metabolize given human milk oligosaccharides [10]. Alternative approaches involve the exposure of infants born by cesarean section to the vaginal microbiota of their mothers [11] or fecal microbiota transplantation (FMT) from healthy donors [12]. The aforementioned approaches aim to shift microbiota composition toward a beneficial composition and have shown promise, but the clinical efficacy and safety of these therapies require further investigation, particularly in the context of premature neonates [11,12].

This review aims to summarize the current research on gut and lung microbiota maturation in preterm infants and the main characteristics of their immature immune systems. In addition, we will discuss the long-term impact of gut microbiota dysbiosis and an immature immune system on the incidence of respiratory diseases later in life. Finally, we will present potential therapeutic strategies targeting gut dysbiosis prevalent in preterm children.

## 2. Mucosal Immunity in Premature Infants

The innate immune system, which develops during gestation, is the first line of defense against pathogens and is composed of different types of structural and innate immune cells, namely neutrophils, dendritic cells (DC), macrophages, monocytes, and natural killer cells (NK). Premature births result in immune deficiencies and a higher risk of developing severe infections and, later in life, respiratory diseases [13]. The inflammatory response of premature infants to microbial agents may be dysregulated for several reasons.

First, the innate immune system of these infants is characterized by low numbers of neutrophils resulting from their reduced output from the bone marrow [14], paired with a compromised ability to migrate to the sites of infection [15]. This could be a consequence of impaired function of the cell adhesion molecules that have been described in the neonatal polymorphonuclear neutrophils (PMNs) isolated from very premature neonates (<32 weeks of GA). The authors reported reduced expression of P-selectin glycoprotein ligand-1 (PSGL-1) and Mac-1 (CD11b), leading to lower recruitment of PMNs and their adhesion capacity after LPS stimulation [16]. Likewise, the basal expression of β2 integrin LFA-1 is also downregulated under basal conditions in preterm PMNs (<37 weeks of GA), and the expression does not increase even after a proinflammatory stimulus, compromising neutrophil recruitment [17]. Another potential factor behind low neutrophil counts is the inadequate production of granulocyte colony-stimulating factor (G-CSF), a regulator of neutrophil function responsible for increasing neutrophil numbers and activity [18]. Furthermore, reduced levels of G-CSF and granulocyte-macrophage colony-stimulating factor (GM-CSF), common in preterm neonates, can result in impaired phagocytosis related to a lack of phagocytic cells [19]. This, together with the lower neutrophil storage pools, leads to neutropenia, which can be detrimental during Gram-negative infections, preceding the development of sepsis [20]. Moreover, phagocytosis is negatively affected in extremely and moderately preterm infants because of deficits in the activation of the complement system and the lack of soluble factors. For example, immunoglobulin G (IgG) is mostly transferred across the placenta after the 32nd week of gestation [21]. IgG levels have been reported to be scarce in extremely preterm infants (<10% maternal levels), and very low in very preterm infants (up to 50% maternal levels) and preterm infants (70–80% maternal levels) when compared to term neonates [22]. Diminished IgG transfer could result from selective neonatal Fc-receptor (FcRn) binding, responsible for capturing antibodies from maternal circulation [23].

Several studies have also reported lower frequencies of NK cells in premature neonates (<37 weeks of GA) [24] and of classical monocytes in moderately premature infants (30.4–34.1 weeks of GA) [25]. Furthermore, NK cells from preterm infants secrete lower levels of chemokines [24]. This may result in deficient DC maturation [26,27] and a biased Th2 phenotype, putting preterm infants at higher risk of developing asthma in childhood [28,29]. However, this predisposition to a Th2 phenotype in the preterm population could also be a consequence of the downregulation of several factors involved in the differentiation of naïve CD4+ T cells, such as the CD40 ligand (CD154), which promotes the differentiation of Th1 cells, or a transcription factor T-bet, which regulates the differentiation of naïve CD4+ T cells into Th1 [30]. Finally, defective production of Th1 cytokines such as IFN-γ could also contribute to the Th2 phenotype in premature neonates [31].

In addition to altered Th1/Th2 balance, Th17 population can also be abnormal in preterm children. Th17 cells play an important role in maintaining homeostasis at mucosal barriers, which are exposed to environmental insults, such as bacteria and fungi. Considering that premature infants are more susceptible to bacterial infections, a diminished Th17 immune response would be expected. However, Black et al. (2012) reported a higher differentiation rate of naïve T cells to a Th17 phenotype in very premature infants (24–31 weeks of GA) than in full-term neonates (>37 weeks of GA) and adults. They showed a higher gene expression of several upstream receptors and transcription factors (*IL-23R*, *STAT3*, *RORC*, *IL6ST*, and *TGFβR1*) in preterm CD4 T cells which are involved in Th17 induction. Increased frequencies of Th17 cells were also reported in the cord blood of preterm neonates, pointing towards an enhanced Th17-mediated inflammatory response [32]. But contrary to typical T cells, in samples collected from preterm neonates, Th17 cells developed from CD161^-^ precursors in vitro [33]. This could point towards deficiencies in producing pro-inflammatory mediators, including IFN-γ and IL-17, compared to typical Th17 cells derived from CD161+ T cells [34]. The reasons behind the apparent discrepancy between increased Th17 population size and high susceptibility to infections remain elusive but might be explained by the impairment in the effector arm of the response, such as neutrophils.

Another subset of T helper cells with profound effects on immunity is the regulatory T cell subset. Interestingly, several studies have reported that the frequency of regulatory T cells (Tregs) is higher in premature infants than in full-term infants during the first days of life, which is inversely correlated with gestational age [35,36]. In line with this, the lymphopenia that characterizes premature infants could be a result of diminished IL-7 production essential for T-cell survival as well as increased Treg population, since this subset of cells inhibits the proliferation of T cells and suppresses their function [36,37]. Increased Treg frequencies during the first 2 weeks of life have been also linked to the development of bronchopulmonary dysplasia (BPD), currently believed to be due to an immunosuppressive environment [36]. BPD-related lung damage, in turn, may increase the probability of developing acute respiratory distress syndrome (ARDS) later in life [38].

Apart from the dysregulated function of neutrophils, another defect in innate immunity that predisposes preterm babies to infection is the impairment in plasmacytoid dendritic cell (pDC) function. For instance, these cells have been described to have a compromised capacity to produce IFN-α [39] and disrupted TLR responses to viral ligands [40]. Such decreased functionality of pDCs would result in impaired antiviral response, potentially leading to more severe or prolonged infections. Moreover, pDC-derived IFN-α has a suppressive effect on airway inflammation and its deficiency can be considered as a susceptibility factor for the development of allergen-induced allergic airway inflammation present in asthma [41]. In addition, the expression level of BDCA-4 (CD304) in this cell type, a surface receptor that allows viral evasion, is also lower in preterm infants compared to adults [39].

Altogether, these data highlight that the immune system of preterm infants may be less effective in combating pathogenic infections, and this will be a risk factor for developing respiratory diseases later in life.

## 3. Gut–Lung Axis in Preterm Neonates

**Figure 1:**
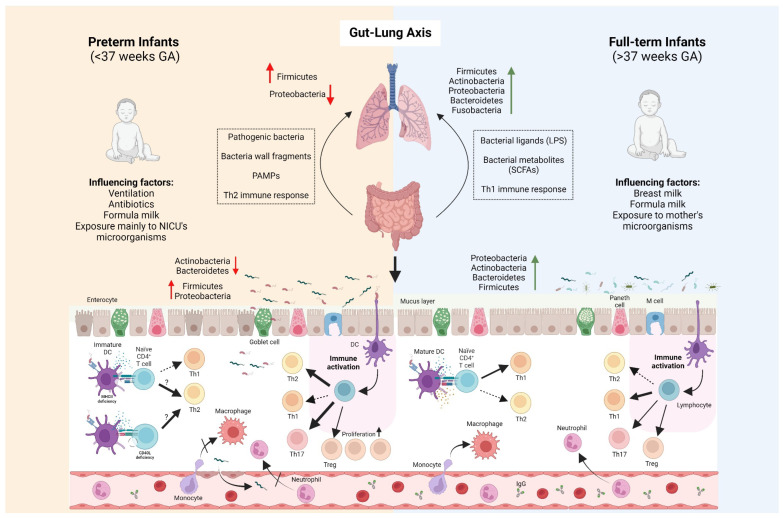
Gut–lung axis in preterm and full-term infants. Several factors such as exposure to antibiotics, NICU’s environment, ventilation and formula-milk feeding influence gut microbiota colonization in preterm infants, changing the microbial composition in respect to full-term infants. Damage to the intestinal epithelial barrier in preterm neonates, along with lower mucus and IgA secretion, allows for the adhesion and translocation of pathogenic bacteria, bacterial fragments, and PAMPs to the circulation, carrying them to distal organs such as the lungs. This, together with the immaturity of their mucosal immune system, normally biased to a Th2 phenotype, can modulate lung immune response and modulate lung microbiota composition, which may increase the susceptibility to respiratory diseases. Under normal conditions, a well-established gut microbiota, together with its secreted metabolites, help to maintain the intestinal barrier and the immune system function, skewed to Th1 phenotype. Similarly, microbial metabolites and immune Th1 response in the gut can also contribute to the maintenance of respiratory homeostasis. Thick and dotted arrows indicate a stronger and weaker bias to a specific phenotype. Question marks denote the unknown/possible relation between elements. Abbreviations: PAMPs: Pathogen-associated molecular patterns; SCFAs: Short chain fatty acids; LPS: Lipopolysaccharide; GA: Gestational age; NICU: Neonatal intensive care unit.

Full-term newborns delivered vaginally are exposed to maternal microbiota in the birth canal while preterm children, more often born by c-section, initially getting in contact with their mother’s skin microbiota and bacteria found in the environment [42]. This is of particular importance since birth marks the beginning of microbial colonization and starts the host–microbiota interactions that set the baby on a trajectory toward health or disease [43,44]. The associations among preterm birth (<37 weeks GA), very low birth weight (<1500 g), and the risk of respiratory outcomes in early life and later in adulthood have been established [45,46,47]. Alterations in the gut microbiota and intestinal physiology are correlated with the occurrence of respiratory disorders later in life through the modulation of the immune response locally and distally in the lungs via the lung–gut axis (Figure 1) [48]. The gut microbiota of healthy neonates predominantly consists of four bacterial phyla as follows: Proteobacteria, Actinobacteria, Bacteroidetes, and Firmicutes [49]. Typically, the gut of newborns is first colonized by facultative anaerobic bacteria, including *Enterobacter*, *Lactobacillus*, and *Streptococcus*, which are later replaced by anaerobic genera such as *Bifidobacterium*, *Bacteroides*, *Clostridium*, and *Eubacterium* [50]. In contrast, preterm children are characterized by abnormal patterns in gut colonization, with a higher abundance of facultative anaerobes and delayed colonization by obligate anaerobes [51,52]. This includes an increased relative abundance of potentially pathogenic genera such as *Enterococcus*, *Enterobacter*, and *Staphylococcus*, coupled with reduced relative abundance of *Bacteroides*, *Bifidobacterium*, and *Atopobium* [53]. The delayed colonization/lower abundance of Bifidobacteriaceae is of particular concern because of their pivotal role in short-chain fatty acid (SCFA) production, which is critical for maintaining homeostasis and human health [54,55]. The hospital environment itself may contribute to the atypical microbiota in preterm neonates. Microbial strains known to cause nosocomial infections, such as *Staphylococcus epidermidis*, *Enterococcus faecalis*, *Pseudomonas aeruginosa*, and *Klebsiella pneumoniae*, were detected in preterm neonates and their respective hospital rooms, indicating environmental microbial contamination [56].

It is well established that the gut microbiome is related to human health, but it is not the only area inside the human body inhabited by microbes. Distinct microbial populations have also been described in the respiratory system. The microbiota present in the airways differs in numbers and composition due to several mechanisms, including migration of microorganisms from the environment, growth rate, elimination of microbes, and local immune responses [57]. The lung microbiota of healthy individuals mainly includes members of the phyla Firmicutes, Actinobacteria, Proteobacteria, Bacteroidetes, and Fusobacteria in the upper respiratory tract, and members of the phyla Firmicutes and Bacteroidetes in the lower respiratory tract [58,59,60]. In the first few days after birth, the respiratory microbiota of infants is dominated by the genera *Staphylococcus* and *Ureaplasma*. Surprisingly, analysis of tracheal aspirates collected during the first days of life showed no significant differences in composition between term and preterm neonates [61]. In contrast, the lung microbiota composition in preterm children suffering from BPD is less diverse and can be distinguished from either the full-term or preterm population not suffering from BPD [62,63]. This perturbed microbiota composition can persist into adulthood. For instance, some studies have reported a less diverse respiratory microbiota in adults born extremely prematurely (<26 weeks of GA), with a reduced abundance of the *Prevotella* genus, an indicator of healthy lung microbiota [64,65]. It is important to note that difficulties in sampling and the risk of contamination may hamper the understanding of airway microbial colonization in neonates. In some studies, dominant bacteria detected in samples of intubated preterm neonates were suggested to originate from the hospital bacterial population [66,67,68].

While chronic respiratory diseases have multifactorial pathogenesis, it is also important to highlight how gut dysbiosis [69] in preterm infants and the immaturity of their immune systems, biased to a Th2 phenotype [70], may increase their susceptibility to respiratory diseases such as asthma and chronic obstructive pulmonary disease (COPD). Several studies have linked a higher incidence of developing these respiratory disorders to prematurity, especially in those children delivered by c-section. For example, in a case-control study conducted with preterm and full-term infants delivered by c-section, the authors showed a higher prevalence of preterm children who were hospitalized with asthma (16.5% vs. 13.2%) between 6 and 12 years of age [71]. Others have also confirmed a greater risk of developing severe asthma [72,73] and COPD [13,45] when the GA decreases compared with full-term infants, with c-section again being one of the factors associated with the severity of the disease. These effects could also be attributed to the immune modulation by the host gut microbiome and/or its derived metabolites. Preterm neonates exhibit impaired intestinal physiology and structure compared to term infants [74], which could be partially due to alterations in gut colonization thus resulting in microbial dysbiosis and imbalanced profiles of associated microbial metabolites [75,76]. For instance, SCFAs serve as an energy source for intestinal epithelial cells and help in maintaining the integrity of the intestinal epithelial barrier [77]. In this context, the translocation of gut-associated bacteria into the lungs of patients suffering from ARDS as well as the alteration of the lung microbiome in mice after sepsis [78], could be a consequence of an increased intestinal permeability due to an imbalance in gut microbiota and its metabolites. On the other hand, intestinal microbiota has a protective effect on the host, for instance during bacterial pneumonia [79] or even during vaccination. A cohort of healthy volunteers with low baseline levels of neutralizing antibodies, and who were treated with antibiotics prior to influenza vaccination (H1N1 A/California strain), displayed reduced concentrations of H1N1-specific IgG1 antibodies. The authors suggested that the disruption of the gut microbiota, mainly characterized by Lachnospiraceae, Enterobacteriaceae and Ruminococcaceae, may negatively affect antibody responses after vaccination [80]. In another study carried out in specific-pathogen-free (SPF) mice, the authors observed that the stimulation via TLR5 by the commensal gut microbiota is important for the optimal antibody responses after influenza vaccination [81]. In addition, other authors have suggested that an imbalance in gut microbiota modulates the response to respiratory viral infections in mice, reporting low numbers of Treg cells and a higher proinflammatory response in the lungs, followed by increased mortality [82]. Despite these data, research focused on premature infants is still needed to fully understand the host–microbe interactions required for the optimal maturation of the immune system and the maintenance of homeostasis.

### Antibiotic Exposure in Preterm Children

Among factors influencing the gut microbiota, the use of antibiotics is one of the most obvious and well documented. In particular, in newborns whose gut microbiota is not yet established, exposure to antibiotics during pregnancy or in early childhood has been linked to an increased risk of childhood allergy, asthma, and weight gain [6,83,84,85,86].

Up to 75% of preterm infants receive at least one dose of antibiotics during their hospitalization after birth, a practice aimed at reducing the morbidity and mortality in this population [87,88]. Apart from using antibiotics for preventing and treating early-life infections, newborns can also be exposed to antibiotics via their mothers during delivery, which has been correlated with negative health outcomes in preterm neonates. For instance, intrapartum antibiotic usage has been associated with an increased incidence of necrotizing enterocolitis (NEC) [89] and sepsis caused by antibiotic-resistant *Escherichia coli* [90]. Antibiotic exposure has also been shown to lower overall microbial diversity in preterm children [91,92]. Strikingly, microbiome dysbiosis caused by antibiotic usage persists after ceasing the treatment. At 30 days, preterm children whose mothers received antibiotics during delivery, had a lower relative abundance of Bifidobacteriaceae and Streptococcaceae but an increased relative abundance of Enterobacteriaceae, compared to the neonates not exposed to antibiotics [93]. Furthermore, preterm newborns treated with antibiotics during their NICU stay, exhibited lower microbiome diversity that persisted up to 40 weeks after concluding the treatment [94]. Beyond short-term consequences, antibiotic exposure is linked to long-term health outcomes, such as allergies and gastrointestinal, neurodevelopmental, or metabolic disorders [95,96,97,98]. However, exact microbe–host interactions that contribute to the above remain unexplored.

## 4. Therapeutic Strategies to Restore Preterm Gut Microbiota

As it has previously been discussed, preterm children are characterized by abnormal colonization patterns of the gut microbiota [52]. On top of less diverse microbiota and more potentially pathogenic strains, preterm infants also have only partially developed mucosal barriers, digestive processes, and immature immune system, all of which relate to microbiota dysbiosis. This makes them more prone to opportunistic infections [99,100] and to the possible development of respiratory diseases later in life [45].

Breastfeeding confers immense health benefits to infants, reducing the risk of NEC and infections in first weeks of life [101,102,103]. While it is still recognized as the golden standard when it comes to feeding newborns, it is harder to establish successful breastfeeding in preterm population; in those cases, alternative treatment options would be greatly beneficial [104,105].

To date, several therapeutic interventions aiming to modify the composition of the gut microbiota in preterm infants have been studied (Table 1). Among those, probiotics and prebiotic supplementation predominate. The effects of probiotic and prebiotic supplementation have already been studied in premature neonates. For example, it has been demonstrated that probiotics given to preterm newborns can not only protect from pathogenic strain growth [7] but also shift microbiota composition towards that of healthy full-term neonates [9,10]. For example, the administration of probiotics/prebiotics containing a mixture of *Lactobacillus*, *Bifidobacterium* species, and fructo-oligosaccharides to premature infants (<35 weeks of GA) shifted their gut microbial composition and accelerated *Bifidobacterium* spp. colonization after 4 weeks [8].

Prebiotics are food components that provide beneficial, often immunomodulatory effects on the host and include oligosaccharides, glycoproteins, glycosaminoglycans, glycolipids, and mucin [106]. High levels of prebiotics are present in human breast milk, and breastfeeding shows a protective effect on NEC and sepsis, along with a reduction in children’s morbidity and mortality [101,102,103]. Human milk oligosaccharides alone confer prebiotic effects by facilitating Bifidobacteria and Lactobacilli growth in the colon of breastfed infants [107,108]. Despite that, since premature infants are normally kept under NICU treatment, they are mainly fed with formula milk. Artificial formulas do not provide such protection as they lack particular components that are present in breast milk, including the human milk oligosaccharides (HMOs). HMO supplementation was reported to improve growth outcomes in preterm population; children fed HMO-enforced formula exhibited increase in length and head circumference statuses, classifiers linked with infant development [109]. Currently, no studies have been carried out to assess the effect of HMO supplementation to restore the microbiota composition in premature neonates. Finally, other studies have shown no significant effects of HMO solitary supplementation of infant formula on NEC prevention [110,111].

A potential preventative measure for gut dysbiosis could be ensuring that newborns get exposed to the mother’s vaginal microbiome. In the case of infants born by c-section, vaginal seeding—soaking a cotton swab with maternal vaginal microbiota and transferring it to the face, mouth, and nose of the newborn—has been gaining interest over the last few years. In a pilot study, vaginal seeding was carried out in infants delivered by c-section and its effect on microbiota composition was assessed. After 30 days, the microbiota of newborns resembled that of vaginally delivered children [11]. Alternatively, another approach could be the oral administration of maternal vaginal microbiota. However, no significant differences have been previously reported in treated infants born by c-section [112,113] and no studies have examined its effect on premature neonates.

If the preventative measures are not sufficient, FMT could pose an attractive treatment option after the gut microbiota dysbiosis is already established. This procedure involves transferring healthy donor microbiota into the recipient’s gastrointestinal tract [12]. A proof-of-concept study showed that FMT mitigated gut microbiota dysbiosis in cesarian-born newborns but also emphasized the need for thorough screenings of the donors so as not to transfer any pathogens [114]. In the case of preterm population, it has only been tested in preterm pigs delivered by c-section. In this animal model, a protective effect against NEC, a lethal bowel disease which is highly prevalent in preterm children related to gut dysbiosis, was demonstrated [115].

Collectively, it is important to better understand current and prospective therapeutic strategies in the context of preterm microbial colonization during the first days of life, which is relevant to future health and prevention of respiratory diseases in adulthood.

**Table 1:** Therapeutic strategies targeting gut microbiota.

Therapeutic Intervention	Findings	Experimental Setup	References
Probiotic supplementation	Shifts microbiota composition towards that of healthy full-term neonates.	NICU-resident preterm infants supplemented with *Bifidobacterium bifidum* and *Lactobacillus acidophilus*. Preterm neonates supplemented with *Lactobacillus rhamnosus* alone or in combination with *Bifidobacterium lactis* Bb-12.	[10,100]
Combination of probiotics and prebiotics	Shifts gut microbial composition and accelerates *Bifidobacterium* spp. colonization after 4 weeks.	Preterm infants supplemented with *Lactobacillus* and *Bifidobacterium* species in combination with fructo-oligosaccharides.	[8]
Prebiotic supplementation	Human milk oligosaccharides supplementation confers prebiotic effects by facilitating Bifidobacteria and Lactobacilli growth in the colon of breastfed infants.	Term infants received milk with a mixture of inulin and galactooligosaccharides.	[108]
Prebiotic supplementation	Increase in length and head circumference statuses.	Preterm infants supplemented with a mixture of 2′-fucosyllactose and lacto-N-neotetraose in a ratio of 10:1. Three portions per day.	[107]
Vaginal seeding	The microbiota of newborns resembled that of vaginally delivered children.	Neonates were swabbed 1 min after delivery with vaginal microbiota on the lips, face, thorax, arms, legs, genitals, anal region and the back.	[11]
Fecal microbiota transplantation (FMT)	Protective effect against NEC.	Rectal, cognate, or oro-gastric FMT administration from healthy piglets to C-section preterm delivered piglets.	[113]
Fecal microbiota transplantation (FMT)	Gut microbiota from CS-born infants resembles that of vaginally delivered neonates.	Term infants received a diluted fecal sample from their mothers, collected 3 weeks prior to delivery.	[114]

## 5. Conclusions

Recent years have brought spectacular advances into the intricate interplay between the immature immune systems of premature newborns and their gut microbiota, emphasizing its implications for long-term respiratory health. Preterm infants face significant challenges in the development of mucosal immunity, with compromised neutrophil function, altered NK cell frequencies, and skewed Th2 responses, rendering them susceptible to infections. The dysregulation in the innate immune response, particularly in pDCs, further contributes to the vulnerability of preterm neonates to infections. The crucial concept of the gut–lung axis in preterm neonates has been highlighted as well. Altered gut microbiota composition, characterized by an overabundance of potentially pathogenic strains and reduced diversity, is linked to an increased risk of respiratory diseases such as asthma and COPD in both childhood and adulthood. The disturbance in the gut microbiome is exacerbated by antibiotic exposure during the neonatal period, which not only compromises microbial diversity soon after but also has lasting effects on long-term health outcomes, including allergies and gastrointestinal disorders.

Several therapeutic strategies have been gaining attention, including probiotic and prebiotic supplementation, exposure to maternal vaginal microbiota, and fecal microbiota transplantation. These approaches aim to restore the composition of the gut microbiota in preterm infants, potentially mitigating the risk of respiratory diseases in the long term. Currently, breastfeeding should still be considered the gold standard, but when not available, prebiotic-supplemented artificial formula should also be considered as preventative strategy to set the developing baby on a trajectory toward health.

While current studies focus on understanding and addressing the challenges faced by preterm infants, continued research is necessary to unravel the complexities of microbial colonization and immune modulation in this vulnerable population. The ultimate goal is to develop effective preventive and therapeutic strategies that not only improve immediate health outcomes but also mitigate the risk of developing respiratory diseases later in life. As advancements in neonatal care continue, a more profound understanding of the intricate relationships between the gut, the lungs, and the immune system will pave the way for targeted interventions, ensuring a healthier future for preterm infants.

## Data Availability

No new data created or analyzed in this study. Data sharing is not applicable to this article.
